# Comparison of the Effects of Coaching and Receipt of App Recommendations on Depression, Anxiety, and Engagement in the IntelliCare Platform: Factorial Randomized Controlled Trial

**DOI:** 10.2196/13609

**Published:** 2019-08-28

**Authors:** David C Mohr, Stephen M Schueller, Kathryn Noth Tomasino, Susan M Kaiser, Nameyeh Alam, Chris Karr, Jessica L Vergara, Elizabeth L Gray, Mary J Kwasny, Emily G Lattie

**Affiliations:** 1 Center for Behavioral Intervention Technologies Northwestern University Chicago, IL United States; 2 Department of Psychological Science University of California, Irvine Irvine, CA United States; 3 Department of Gastroenterology Northwestern University Chicago, IL United States; 4 Audacious Software Chicago, IL United States; 5 Department of Preventive Medicine Northwestern University Chicago, IL United States

**Keywords:** depression, anxiety, mHealth, clinical trial

## Abstract

**Background:**

IntelliCare is a modular platform that includes 12 simple apps targeting specific psychological strategies for common mental health problems.

**Objective:**

This study aimed to examine the effect of 2 methods of maintaining engagement with the IntelliCare platform, coaching, and receipt of weekly recommendations to try different apps on depression, anxiety, and app use.

**Methods:**

A total of 301 participants with depression or anxiety were randomized to 1 of 4 treatments lasting 8 weeks and were followed for 6 months posttreatment. The trial used a 2X2 factorial design (coached vs self-guided treatment and weekly app recommendations vs no recommendations) to compare engagement metrics.

**Results:**

The median time to last use of any app during treatment was 56 days (interquartile range 54-57), with 253 participants (84.0%, 253/301) continuing to use the apps over a median of 92 days posttreatment. Receipt of weekly recommendations resulted in a significantly higher number of app use sessions during treatment (overall median=216; *P*=.04) but only marginal effects for time to last use (*P*=.06) and number of app downloads (*P*=.08). Coaching resulted in significantly more app downloads (*P*<.001), but there were no significant effects for time to last download or number of app sessions (*P*=.36) or time to last download (*P*=.08). Participants showed significant reductions in the Patient Health Questionnaire-9 (PHQ-9) and Generalized Anxiety Disorder-7 (GAD-7) across all treatment arms (*P* s<.001). Coached treatment led to larger GAD-7 reductions than those observed for self-guided treatment (*P*=.03), but the effects for the PHQ-9 did not reach significance (*P*=.06). Significant interaction was observed between receiving recommendations and time for the PHQ-9 (*P*=.04), but there were no significant effects for GAD-7 (*P*=.58).

**Conclusions:**

IntelliCare produced strong engagement with apps across all treatment arms. Coaching was associated with stronger anxiety outcomes, and receipt of recommendations enhanced depression outcomes.

**Trial Registration:**

ClinicalTrials.gov NCT02801877; https://clinicaltrials.gov/ct2/show/NCT02801877

## Introduction

### Background

Depression and anxiety are common mental health problems that impose a very high societal burden in terms of cost, morbidity, quality of life, and disability worldwide [1-4]. Most people experiencing these common mental health problems cannot access treatment because of a variety of barriers including the lack of availability of services, time constraints, transportation problems, and high cost [5,6]. A wide variety of digital mental health interventions have demonstrated efficacy. Adherence and outcomes appear to be stronger when coupled with human coaching through telephone or messaging than in self-guided digital interventions [7-9]. Mobile apps, in particular, have a number of advantages. Smartphones are becoming ubiquitous in developed countries and are increasingly common in developing nations [10]. As people keep their phones with them, app-based interventions can fit more seamlessly into the fabric of people’s lives.

Most digital interventions for depression or anxiety are single Web-based and mobile apps. Very few of the publicly available apps have been rigorously tested, with reviews suggesting that the percentage of available apps for depression and anxiety with any evidence of effectiveness may be around 2.6% to 3.8% [11,12]. A common design approach is to adapt an effective psychotherapeutic model such as cognitive behavioral therapy (CBT) and digitize it into an app format. Such apps typically contain a multitude of features related to the treatment model on which they are based. For example, in CBT, these apps might contain psychoeducation, symptom tracking, activity monitoring, activity scheduling, and cognitive restructuring [13]. This approach of feature-rich apps does not recognize how most people currently tend to use apps and their smartphones. In general, digital technologies have core features that break into basic components, allowing users to piece together those components that are most useful. For example, people tend not to have 1 app to meet their transportation needs; rather, they usually have multiple apps that search for locations and map routes, and different apps for different modes of transportation. Different people likely use different apps according to their preferences and lifestyles. Typically, popular apps serve singular purposes, such as searching for restaurants or businesses, managing flights, or posting pictures. People tend to use apps in very short bursts of time, and sometimes frequently [14,15]. Thus, apps tend to support a single or limited set of related tasks through simple, quick interactions. Indeed, even when people do use existing mental health apps, they typically only use 1 or 2 features. For example, in an evaluation of PE Coach, which contains a number of features, it was found that clinicians and patients mostly used the app to audio-record sessions [16]. This gap between the design of mental health apps, which is typically based on complex psychotherapy models, and how people use their devices, which commonly occurs in short bursts for single purposes, likely is 1 reason for the low engagement with mental health apps seen in real-world settings [17].

The IntelliCare platform was designed to address user engagement problems by providing self-help strategies and skills training in a manner that is consistent with how people use mobile phones. Rather than a single app containing a comprehensive set of behavioral strategies, the IntelliCare platform currently comprises 12 apps, each of which is focused on a single psychological or behavioral strategy. The time required for each use is short, with most uses lasting less than 1 min [18]. Thus, apps can be integrated more seamlessly into a person’s life. Users are able to select and use apps that they find helpful and ignore the ones they do not like. A Hub app, if downloaded, coordinates the user’s experience and provides weekly recommendations to try new apps. The provision of recommendations appears to be an important component in maintaining engagement with the IntelliCare platform. Indeed, the receipt of these recommendations has been shown to increase the likelihood that an individual will download the recommended app [19,20].

IntelliCare apps have been available on the Google Play Store, beginning in 2014, and have been downloaded more than 100,000 times. An initial field trial, in which participants received 8 weeks of coaching primarily through text messages, showed substantial improvements in both depressive and anxiety symptoms’ severity, and strong, consistent engagement of an average of 3 to 4 app launches each day over the full 8 weeks [18]. Interestingly, although the coached field trial showed participants used most of the apps, the average use within any individual app was substantially different from that observed among users who simply downloaded the apps through the Google Play Store, suggesting that coaches may have encouraged exploration of new apps but did not appear to influence continued use once users had downloaded and tried the apps. Thus, there appear to be 2 methods of encouraging exploration in app platforms such as IntelliCare: coaching and automated recommendations.

Although sustained behavioral engagement, or app usage, has been noted to be a major problem in digital mental health interventions [21,22], few investigations have systematically explored different methods of maintaining engagement. Moreover, 1 study explored a factorial design of 5 different engagement elements, including automated versus human support, text messages, tailoring of success stories, personalization of content, and multimedia and interactive materials [23]. Human support was the only element that improved outcomes during the intervention period, which is consistent with a large body of literature showing human support improves engagement and outcomes [7,24]. However, those who received automated support experienced more change during the postintervention period. Given the ubiquity of app store recommendations, this is a promising element to evaluate.

### Objectives

This study aimed to examine the effect of 2 separate methods of maintaining engagement with the IntelliCare platform: coaching and receipt of weekly recommendations to try different apps. We hypothesized that coaching would produce better engagement with the apps and greater reductions in symptoms of depression and anxiety than that associated with self-guided use, and those who received both coaching and app recommendations would have the greatest reductions in symptoms and highest use of apps.

## Methods

### Participants

Participants were recruited from July 5, 2016, to May 5, 2017, through a variety of digital (eg, Instagram, Facebook, and Reddit) and print (eg, advertisements on Chicago Transit Authority bus and train lines) sources as well as research registries (eg, ResearchMatch), commercial recruitment firms (eg, Focus Pointe Global), and media coverage using methods that have been previously described [25,26]. Participants were included if they met criteria for depression (Patient Health Questionnaire-9 [PHQ-9]≥10) [27] or anxiety (Generalized Anxiety Disorder-7 [GAD-7]≥8) [28], were aged 18 years or older (aged 19 years if in Nebraska, given age of consent), resided in the United States, could speak and read English, and had an Android phone with data and text plans. Participants were excluded if they (1) had visual, voice, motor, or hearing impairments that would prevent participation; (2) met diagnostic criteria for a severe psychiatric disorder such as psychotic or bipolar disorders for which study treatments would be inappropriate; (3) imminent suicidality that included both a plan and intent; (4) had initiated or modified antidepressant pharmacotherapy in the previous 14 days; or (5) had used any IntelliCare app more than 1 time in the 3 months before study screening.

All procedures were approved by the institutional review board of Northwestern University. Participants completed a Web-based consent form, and a research assistant reviewed the Web-based consent document to ensure comprehension questions were answered correctly and that the consent form was signed. Any questions or concerns were then reviewed with participants. The trial was monitored by an independent data safety monitoring board.

### Treatments

This trial used a 2 × 2 factorial design (coached vs self-guided treatment and weekly app recommendations vs no recommendations), resulting in 4 treatment cells. A no treatment or waitlist control was not included because it would be impossible to prevent control participants from accessing the apps, which are freely available on the Google Play Store, and it could not be reliably determined which apps were accessed and when. The IntelliCare platform, coaching protocol, and the recommended system are described below.

#### IntelliCare Platform

All participants received access to the IntelliCare platform apps. At the time of this trial, the IntelliCare platform consisted of 13 apps [18,20]. This included 12 clinical apps, each of which was designed to target a specific behavioral or psychological treatment strategy (eg, goal setting, behavioral activation, social support, living one’s values, cognitive restructuring, emotion regulation, positive self-affirmations, coping, exercise for mood, sleep hygiene, relaxation, and psychoeducation with reminders) and improve symptoms of depression and anxiety through efficacious treatment strategies. Most apps were designed to require less than 30 seconds to use. Apps included automated reminders to encourage engagement and for both app use and implementation of the strategies. The user’s experience with the clinical apps was coordinated through a Hub app that consolidated automated notifications and provided app recommendations for those who were randomized to receive recommendations. No substantive changes were made to the apps during the course of the trial.

#### Coaching Versus Self-Guidance

Coaching was guided by the IntelliCare coaching manual [29], which is based on the supportive accountability model [30] and the efficiency model [31], and was aimed primarily at encouraging participants to try the apps, answering questions about how to use the tools represented in the apps and the rationale behind the skills taught by the apps, encouraging application of the skills in daily life, and providing some technical support as needed. Coaching began with an initial 30- to 45-min engagement phone call to explain the program, understand the participant’s goals for mood and anxiety management, set expectations for the coach-participant relationship, build rapport, and ensure the Hub app was properly installed on the participant’s phone. After the initial engagement call, participants received 2 to 3 text messages per week from their coach to provide support in using apps, offer encouragement, reinforce app use, and check-in on progress or challenges. Coaches also responded to all participant-initiated text messages within 1 working day. Coaches offered but did not require an additional 10-min call around midtreatment to support engagement. The coaches had a dashboard that provided information about the IntelliCare apps on each participant’s phone, including which apps were installed, when they were downloaded, each time an app was used, and which apps were selected as *primary* in the Hub app. The dashboard also included a short message service text messaging tool, a section for brief notes, and an alert indicating when no IntelliCare app had been used for 3 days or when a participant sent a text message indicating they might be at risk for self-harm, which resulted in an automated safety response and prompted coaches to check-in. Coaches had at least a bachelor’s degree in psychology or a related field and were trained and monitored by 1 of the coaching manual authors.

Participants assigned to self-guidance received the initial 10- to 15-min engagement call to ensure the Hub app was properly installed and that they understood how to use the IntelliCare platform but had no further contact with coaches.

#### Recommendations

Participants randomized to the recommendation arm received a weekly tray notification on their phone’s home screen, which took them to the Hub app, where they were provided with weekly recommendations for new apps. Touching the recommendation button took them to the app store where they could download the recommended app. The recommendation engine used app usage data from approximately 100,000 users who had downloaded the IntelliCare apps from the Google Play Store to identify, based on the individual user’s app use profile, apps that the individual was more likely to use. Participants were asked to at least to try the newly recommended apps but were encouraged to use the apps they found most helpful.

Those participants who were in the no recommendation arm had the recommendation feature in the Hub app removed and were simply encouraged to explore the apps on the IntelliCare platform.

### Outcome Assessment

Depression was measured using the PHQ-9 [27], and anxiety was measured using the GAD-7 [32]. These measures were administered as Web-based self-reports through Research Electronic Data Capture [33] at baseline, week 4, week 8 (end of treatment), and 3- and 6-month follow-up and completed by the participants themselves.

Engagement was defined using 3 commonly used behavioral engagement metrics [34]: *time to last use, number of app sessions*, and *number of apps downloaded*. Number of app sessions is a very common metric. *Time to last use* was defined as the time between the first launch and the last launch of any app during the 8-week trial or posttrial period. Posttrial app use data were truncated at 6 months to avoid biases related to time of entry into the trial. *Number of app sessions* is a commonly used metric*.* In this study, an app use session was defined as a sequence of user-initiated actions or events separated by less than 5 min between events. A new app launch (or session) was defined as a new activity after 5 min of no activity (we note that some apps have audio or video content that may last longer than 5 min, in which case, the running content is counted as activity). *Number of apps downloaded* is similar to the number of features or modules used in feature-rich applications [34]. Given the IntelliCare unbundled features into individual apps, this was defined here as the number of apps downloaded with at least one launch.

### Randomization and Masking

A statistician provided a sequentially masked randomization scheme, created before the start of the trial, assigning participants to (1) coached with recommendations, (2) coached without recommendations, (3) self-guided with recommendations, and (4) self-guided without recommendations, stratified by current antidepressant medication status and psychotherapy status, with a block size of 4 within each stratum.

### Statistical Analyses

Power was calculated on the assumption that approximately 50% of patients stop using the apps by week 7 based on previous electronic health work [35]. We powered for an effect size of 0.30 or a difference between 50% and 61.6% between groups. On the basis of a type I error rate of 5% and 80% power, power calculations using a log-rank test indicated a required sample size of 135 per arm (total sample of 270). Assuming 15 participants would be lost to follow-up in each arm, we aimed to recruit 150 participants in each arm. This provided power to detect effect sizes of 0.34 for clinical outcomes based on independent *t* tests. Week 7 was selected, rather than week 8, to avoid any *end-of-treatment* effects that might occur, such as participants ceasing or reinitiating engagement as a result of approaching end of treatment. Power calculations were performed using PASS 2008. No power calculations were performed to determine effect sizes detectable when examining the relationship between use metrics and patient-centered outcomes, as those were secondary aims.

Descriptive statistics are provided for baseline demographic variables across the 4 groups. Log-rank tests were performed to determine if the time to study dropout was different for each of the main effects, Kaplan-Meier plots are presented, and the engagement rate at 7 weeks. Cox proportional hazard models were used to compare the main effects while adjusting for randomization strata, medication use, age, and sex. Mean and standard deviation for PHQ-9 and GAD-7 over time and randomization groups are presented. Generalized linear mixed models were used to compare patient outcomes, adjusting for randomization strata, and baseline values of PHQ-9 or GAD-7, and assuming a heterogeneous unstructured covariance structure by randomization strata. First, 3-way interactions between time and the main effects of the 2X2 factorial were tested. If those were not significant, 2-way interactions with each main effect and time were modeled and, subsequently, models with main effects without the interaction. If interactions were not significant, within the main effect of time, least square means and differences adjusting for randomization strata, relative to baseline, were tested using Dunnett adjustment for multiple comparisons. Owing to the skewed nature of use data, app launches and time to last use were compared using nonparametric Kruskal-Wallis tests when comparing all 4 groups and Wilcoxon rank-sum test if comparing 2 groups. The relationship between use data and treatment outcome was examined using linear models adjusting for baseline PHQ-9 or GAD-7 and randomization strata. All analyses were run in R version 3.5.1 or SAS version 9.4 [36].

## Results

### Participants

**Figure 1:**
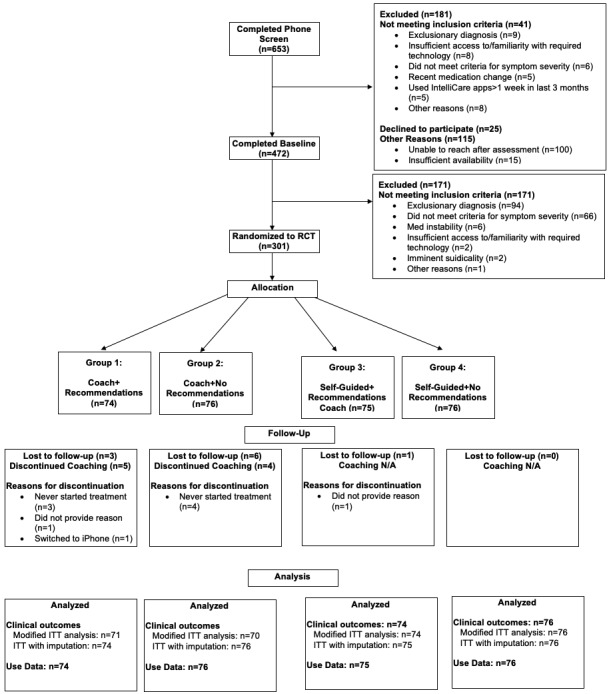
Flow of participants through the trial.

The flow of participants through the study is depicted in Figure 1. Lost to follow-up rates differed significantly across treatment cells (*P*=.02). Table 1 summarizes the baseline demographics and psychiatric characteristics of the participants.

**Table 1:** Participants’ characteristics.

Characteristics		Coached or recommendations (N=74)	Self-guided or recommendations (N=75)	Recommendations or coached (N=76)	No recommendations or self-guided (N=76)
Age (years), mean (SD)		37.57 (12.22)	36.17 (11.49)	37.09 (12.28)	35.34 (11.46)
**Gender, n (%)**					
	Female	57 (77)	54 (72)	62 (81)	55 (72)
	Male	15 (20)	21 (28)	14 (18)	21 (27)
	Other	2 (2)	0 (0)	0 (0)	0 (0)
**Race, n (%)**					
	White	57 (77)	63 (84)	57 (75)	60 (78)
	Black	10 (13)	5 (6)	8 (10)	6 (7)
	Asian	3 (4)	0 (0)	5 (6)	2 (2)
	Other	4 (5)	7 (9)	6 (7)	8 (10)
**Ethnicity, n (%)**					
	Non-Hispanic	62 (83)	67 (89)	68 (89)	71 (93)
	Hispanic	10 (13)	8 (10)	7 (9)	5 (6)
	Missing	2 (2)	0 (0)	1 (1)	0 (0)
Insurance=yes, n (%)		69 (93)	62 (82)	73 (96)	70 (92)
**Marital status, n (%)**					
	Married/partnered	36 (48)	36 (48)	36 (47)	43 (56)
	Single	30 (40)	29 (38)	31 (40)	26 (34)
	Separated/divorced/widowed	8 (10)	10 (13)	9 (11)	7 (9)
**Education, n (%)**					
	High school or less	4 (5)	4 (5.3)	4 (5)	3 (3)
	Some college	16 (21)	18 (24)	11 (14)	15 (19)
	College degree	53 (71)	53 (70)	61 (80)	58 (76)
	Missing	1 (1)	0 (0)	0 (0.0)	0 (0.0)
Household income median, US$ (IQR^b^)		58,000.00 (39,000.00-100,000.00)	50,000.00 (27,000.00-80,000.00)	60,000.00 (32,000.00-92,000.00)	57,500.00 (37,750.00-100,000.00)
Antidepressant status=yes, n (%)		35 (47)	31 (41)	37 (48)	37 (48)
Baseline GAD-7^c^, mean (SD)		11.86 (4.05)	11.88 (3.85)	12.33 (4.51)	11.84 (3.66)
Baseline PHQ-9^d^, mean (SD)		12.78 (4.45)	13.24 (4.55)	13.11 (4.75)	13.70 (4.79)

^a^IQR: interquartile range.

^b^GAD-7: Generalized Anxiety Disorder-7.

^c^PHQ-9: Patient Health Questionnaire-9.

### Engagement Outcomes

#### Time to Last Use

**Figure 2:**
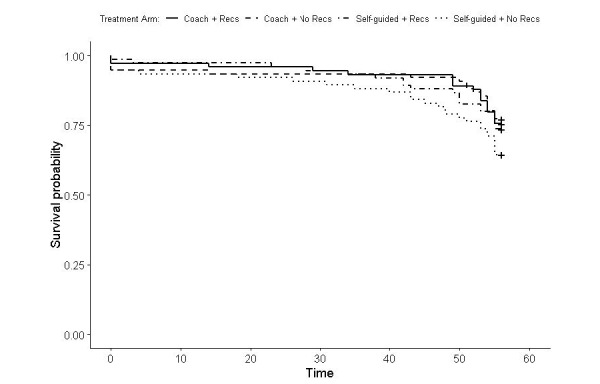
Survival analysis: time to last app launch by treatment cell. Recs: recommendations.

The median time to last use of any app was 56 days (IQR 54-56). Figure 2 shows the Kaplan-Meier estimates for the time to last engagement by the groups. There was no significant difference in coached versus self-guided treatment (log-rank *P*=.94), and the difference between recommendation versus no recommendation did not reach significance (log-rank *P*=.06). The mean engagement percentages and 95% CIs for the number of last uses at 7 weeks or after for the coached and noncoached treatment were 90.7% (86.1%-95.4%) and 83.4% (77.7%-89.6%), and for recommendations versus no recommendations were 88.6% (83.6%-93.8%) and 85.5% (80.1%-91.3%), respectively.

**Table 2:** Median (interquartile range) app sessions by week and treatment arm.

Week	Coached	Self-guided	Recommendations	No recommendations	All treatments
1	19 (12-27)	21 (7-46)	19 (9-29)	21.5 (11.75-40)	20 (10-35)
2	24.5 (14-38.5)	27 (11-43)	24 (13-40)	27 (13.75-43)	25 (13-41)
3	29 (15-44)	27 (11-42)	27 (12-46)	28 (13.75-40.25)	28 (13-43)
4	29 (14-44)	27 (14-40.5)	29 (16-51)	24.5 (12.75-38)	29 (14-43)
5	30 (13.25-50.75)	26 (11-42)	29 (13-49)	27 (11-41.25)	28 (13-45)
6	32.5 (14.25-49.75)	23 (11-40)	34 (14-54)	22.5 (12.75-39.25)	27 (13-46)
7	28.5 (12-43.75)	20 (7-38)	33 (12-53)	19 (8.75-35)	23 (9-42)
8	27 (11-40)	19 (5.5-39)	29 (9-48)	17.5 (7-34)	22 (8-40)
Total	215 (141-330.75)	218 (113-310)	232 (126-356)	201.5 (125.75-285.5)	216 (126-319)

#### Number of App Sessions

Table 2 displays the number of app launches by week across treatment arm. The median number of app sessions was 216 (IQR 126-325) across all apps. There was a significant effect for recommendations, with those receiving app recommendations having a median of 232 (IQR 126-356) app sessions versus those who did not receive recommendations, who had a median of 202 (IQR 126-286) app session (*P=*.04). There was no significant effect for coaching on number of app sessions (coached median 215 [IQR 141-331]; self-guided median 218 [IQR 113-310]; *P*=.36).

#### Number of Apps Downloaded

Participants who received coaching downloaded a median of 11 apps (IQR 10-12), whereas those who did not receive coaching downloaded a median of 7 apps (IQR 4-10), a difference that was statistically significant (Wilcoxon *P*<.001). The effect of receipt of recommendations on number of apps used did not reach significance (Wilcoxon *P*=.08).

#### Use Data During 6-Month Follow-Up

After completion of the trial, 253 (84.0%, 253/301) participants continued using the IntelliCare apps. Among those who continued to use the apps, the median time from the end of treatment to last use was 92 days (IQR 14-178), with a median of 83 (IQR 11-286) sessions. Neither length of use nor number of app sessions varied by treatment group (χ^2^_3_=1.4, *P*=.72; χ^2^_3_=0.3, *P*=.97, respectively).

### Depression and Anxiety Outcomes

Table 3 displays the unadjusted outcomes across treatment groups. Participants showed significant reductions in the PHQ-9 and GAD-7 over time across both treatment arms (*F*_3,844_=16.8, *P*<.001; *F*_3,844_=16.8, *P*<.001, respectively).

Coached treatment produced significantly larger reductions in the GAD-7 than self-guided treatment (*F*_1,844_=4.97; *P*=.03); however, there was no interaction between coaching and time (*F*_3,841_=0.32; *P*=.81). The benefits of coaching did not reach significance for PHQ-9 (*F*_1,844_=3.59; *P*=.06), and there was no evidence of an interaction with time (*F*_3,841_=0.81; *P*=.49).

There was a significant interaction between receiving recommendations and time for the PHQ-9 (*F*_3,841_=2.73; *P*=.04), such that those who received recommendations showed stronger improvements (*F*_3,841_=14.56; *P*<.001) than those who did not (*F*_3,841_=5.09; *P*=.002). Simple effects for recommendations on PHQ-9 are not reported, given the significant interaction effects. There was no significant effect of receiving weekly app recommendations on the GAD-7 (*F*_1,844_=0.05; *P*=.82) and no significant interaction with time (*F*_3,841_=0.65; *P*=.58).

There was no significant interactive effect of coaching and receiving recommendations over time for either the PHQ-9 or GAD-7 (*F*_3,835_=0.19, *P*=.90; and *F*_3,835_=0.73, *P*=.53, respectively).

**Table 3:** Unadjusted means (standard deviation) for the Patient Health Questionnaire-9 and Generalized Anxiety Disorder-7.

Treatment arm		Baseline	Week 4	Week 8	After 3 months	After 6 months
**PHQ-9^a^**						
	Coached	12.95 (4.59)	8.81 (5.30)	7.17 (5.36)	7.03 (5.03)	6.93 (5.58)
	Self-guided	13.47 (4.67)	9.56 (5.01)	8.43 (4.75)	8.45 (5.15)	8.32 (5.32)
	Recommendations	13.01 (4.49)	8.81 (5.3)	7.51 (5.15)	7.41 (4.99)	7.74 (5.77)
	No recommendations	13.40 (4.77)	8.99 (4.95)	8.13 (5.01)	8.12 (5.27)	7.56 (5.19)
**GAD-7^b^**						
	Coached	12.1 (4.28)	7.86 (4.64)	6.76 (4.80)	6.26 (4.77)	6.24 (4.65)
	Self-guided	11.86 (3.75)	8.34 (4.6)	7.45 (4.5)	7.19 (4.59)	6.99 (4.85)
	Recommendations	11.87 (3.94)	8.3 (4.64)	7.06 (4.56)	6.65 (4.67)	6.66 (4.95)
	No recommendations	12.09 (4.1)	7.91 (4.61)	7.17 (4.75)	6.83 (4.73)	6.59 (4.59)

^a^PHQ-9: Patient Health Questionnaire-9.

^b^GAD-7: Generalized Anxiety Disorder-7.

#### Secondary Analysis Using Multiple Imputation

As there was a small but statistically significant difference in the lost to follow-up rate, we conducted a secondary analysis using the expectation-maximization algorithm to impute 5 distinct datasets, in which 4-week outcomes were imputed for any participant who did not have at least one follow-up assessment. This allowed all participants to be included in our generalized linear mixed models. Parameter estimates and corresponding standard errors from each of the 5 models were combined and included in the SAS MIANALYZE procedure to derive valid inferences for the parameters of interest. Conclusions drawn from those analyses were consistent with those presented in our results above, namely, the interaction of the recommendation system and time for PHQ-9 (*P*=.04), the effect of coaching on GAD-7 (*P*=.02), and the changes in PHQ-9 and GAD-7 over time (*P* s<.001).

### Relationship Between App Use and Outcomes

End of treatment PHQ-9, controlling for baseline, was significantly related to the number of app sessions (beta=−.01; *P*<.001), time to last use (beta=−.09; *P*=.001), and number of apps downloaded (beta=−.26; *P*=.001). GAD-7 outcome, controlling for baseline, was significantly related to the number of app downloads (beta=−.16; *P*=.03), but the effect did not reach significance for number of app sessions (beta=−.003; *P*=.05) and time to last use (beta=−.04; *P*=.08) did not reach significance. There were no significant interaction effects for treatment arm and PHQ-9 with number of app sessions (*P*=0.26), time to last use (*P*=0.70), or number of downloads (*P*=0.69). Similarly, there were no signification interaction effects for treatment arm and GAD-7 with number of app sessions (*P*=0.49), time to last use (*P*=0.77), or number of downloads(*P*=0.64).

## Discussion

### Principal Findings

Participants using the IntelliCare app platform showed substantial reductions in symptoms of depression and anxiety, similar to effects previously observed [18]. Coaching resulted in significantly lower levels of anxiety relative to self-guided treatment; however, the effect of coaching on depression was only marginal (*P*=.06). Although there was a difference between depression and anxiety in whether the criterion for significance was met, both *P* values were close to the .05 cutoff, and thus, there was no meaningful difference in the effect of coaching on depression versus anxiety. Receiving weekly recommendations resulted in significantly greater reductions in depression than not receiving recommendations, but there was no similar effect for anxiety. This difference was large. We speculate that the recommendations are more useful for people with depression, as they address motivational challenges faced by people with depression.

App use was strong, with a median of 216 app sessions per participant (an average of 3.9 sessions per day) and a median last day of use being day 56 of 56 days of treatment, with no substantial change in the rate of use over the 8 weeks. Furthermore, 84.0% (253/301) of the participants continued using the apps for a median of 92 days after the completion of the 8-week treatment. This high level of engagement stands in stark contrast to most digital mental health apps, which tend to show sharp drop-offs in the first weeks [7,37]. This is likely because of 2 factors. First, the novelty of having new apps to use over the course of an 8-week treatment likely increases engagement [38]. Second, most of the apps are brief, requiring less than 30 seconds to use, allowing users to fit them into the context of their lives [18]. This suggests that the strategy of providing a platform of simple apps that patients can integrate into the fabric of their lives elicits stronger engagement than more traditional forms of digital mental health that are based on psychotherapy models and require greater time commitments.

People who received weekly recommendations to try new apps engaged in significantly more app sessions, compared with those not receiving recommendations. There was a similar trend for an effect of weekly recommendations on number of apps downloaded and time to last use, although these did not reach significance. These findings are generally consistent with findings of studies of IntelliCare downloads from the Google Play Store, in which users who had installed the Hub app and received recommendations were more likely to download recommended apps and used them more frequently, compared with those who did not download the Hub app and therefore did not receive recommendations [19,20]. These findings support the idea that providing recommendations for new apps on a regular basis can promote behavioral engagement with an app platform.

Participants in the coached conditions downloaded more apps than did those who were self-guided; however, there were no effects of coaching on any other use metrics. This suggests that coaches can help people stay engaged with the platform by trying new apps. However, we speculate that once an app is on the person’s phone, the user’s determination as to whether it is of sufficient value to continue using it is less modifiable by coaches, at least with the present coaching model.

Although the effect of coaching on anxiety and depression was significant or marginally significant, the effect sizes were smaller than we had expected. This stands in contrast to a number of meta-analyses that have consistently shown coaching to have a strong effect [8,39]. There are a number of potential reasons for our weaker than expected findings for coaching. First, the uncoached participants did have an initial 10-min call with a coach to ensure the app was properly installed and that the person knew how to engage with the platform. This may have provided some motivation and reduced confusion that could have led to nonengagement. It is also possible that the automated reminders to use the apps that are part of each app’s design fulfilled some of the coaches’ function in encouragement. However, such reminders are common features of mobile apps, and thus, we expect these automated reminders alone do not fully account for the weak coaching effects. The weaker than expected effects of coaching may be because of the strong app usage observed in this study. The design of the IntelliCare platform emphasized the usability of apps over the application of a theory-based approach, such as modeling the design of an app on CBT. Apps were designed to be simple and quick to use, thereby fitting into the fabric of users’ lives. Coaches have often been employed to encourage the use of intervention technologies. It may be that the coach’s role of encouraging adherence becomes less important as we improve the usability of the apps. Although coaches will likely continue to be beneficial for some people using the IntelliCare platform, these findings suggest that better design of technologies may limit the need for human support, thereby increasing their scalability.

The relationship between engagement metrics and outcomes was mixed, with number of app use sessions being significantly related to both depression and anxiety outcomes, but time to last use and number of app downloads were only related significantly to depression outcomes and not anxiety. However, consistent with much of the literature, even where relationships were significant, they were not strong [40]. There are a number of potential reasons for this. One is that the engagement metrics were strong with fairly high consistency, and thus, the weaker findings may be an artifact of this low variability. However, it is also likely that the relationship between these behavioral engagement metrics and overall symptom change during the intervention obscures more complex relationships. For example, although engagement may reduce symptoms, higher symptoms may increase engagement in the immediate time frame [35]. Thus, simple associations between overall engagement and symptom reduction over the course of treatment may obscure more complex relationships over shorter time frames. Another problem may be that behavioral engagement metrics do not capture meaningful engagement. Indeed, the field of human-computer interaction has viewed engagement more holistically than psychology, considering not only behavioral metrics but also many other subjective factors related to the user’s cognitive and emotional engagement with the apps and intervention [41,42]. This richer conceptualization of engagement may provide a richer understanding of the user’s experience and thus may be more strongly related to clinical outcomes relative to metrics that rely solely on app usage data.

### Limitations

This study has a number of limitations that should be considered in the interpretation of these results. First, lost to follow-up rates were slightly albeit significantly greater in the coached arms than in the noncoached. It is notable that most of those in the coaching arms who were lost to follow-up never initiated treatment, suggesting a small number of people may prefer uncoached interventions. Nevertheless, this difference in lost to follow-up rates across the treatment cells likely did not impact the findings, as it was very small (9% in arm with the highest rate and 3% overall), and secondary analyses imputing missing values showed no difference in outcomes. Second, as a research study, participants had to go through the usual consenting and screening procedures and agree to regular follow-up assessments. This likely resulted in a sample that was more motivated to engage in digital mental health treatment than the average person with depression or anxiety. Thus, the robust level of engagement seen in this sample may not be strong in real-world treatment settings. It is possible that strategies to support engagement (ie, coaching and recommendations) may be more important for less motivated groups. Similarly, we did not conduct diagnostic evaluations and therefore cannot determine how this sample may or may not be similar to populations in health care settings. Finally, this trial did not control for receipt of the IntelliCare apps. Thus, although the reductions in depression and anxiety are substantial, we cannot rule out the possibility that these changes are because of factors other than the treatment, such as the natural course of the symptoms, user expectancies, or research procedures such as repeated assessment.

### Conclusions

This study explores a new paradigm in digital mental health interventions. IntelliCare moves away from the single app for a mental health problem and recognizes 1 of the basic properties of digital tools—that they are broken down into their smallest, simplest elements possible, thereby allowing people to bundle them as they see fit [43]. The strong engagement in IntelliCare suggests that this principle also applies to digital mental health interventions and tools. Engagement with the platform is enhanced through weekly recommendations to try new apps. There is some support for the use of coaches to enhance anxiety outcomes and recommendations to enhance depression outcomes. This suggests that coaching may not be necessary for all people using modular, platform-based digital mental health treatments such as IntelliCare.

## Abbreviations

CBT: cognitive behavioral therapy
GAD-7: Generalized Anxiety Disorder-7
IQR: interquartile range
PHQ-9: Patient Health Questionnaire-9
